# 0427. Respiratory effects of noisy ventilation depend on the etiology of acute respiratory distress syndrome

**DOI:** 10.1186/2197-425X-2-S1-P25

**Published:** 2014-09-26

**Authors:** L Moraes, C Samary, RS Santos, DS Ornellas, CL Santos, NS Felix, R Huhle, P Pelosi, M Gama de Abreu, PL Silva, PRM Rocco

**Affiliations:** Laboratory of Pulmonary Investigation, Carlos Chagas Filho Biophysics Institute, Federal University of Rio de Janeiro, Rio de Janeiro, Brazil; Department of Anesthesiology and Intensive Care Therapy, University Hospital Carl Gustav Carus, Dresden, Germany; IRCCS AOU San Martino-IST, Department of Surgical Sciences and Integrated Diagnostics, University of Genoa, Genoa, Italy

## Introduction

Healthy biological systems are characterized by intrinsic variability of their function, including the respiratory system. However, during pathological conditions, as observed in acute respiratory distress syndrome (ARDS), this biological variability can be lost, and the institution of variable ventilation (noisy, VV) may lead to morphofunctional improvement compared with conventional ventilation (CV). However, it is not known if this beneficial effect is dependent on the etiology of ARDS. In pulmonary ARDS (ARDSp) there is a predominance of lung tissue consolidation, whereas extrapulmonary ARDS (ARDSexp) is associated with alveolar collapse.

## Objectives

The aim of the present study was to compare variable ventilation with conventionalventilation in experimental ARDSp and ARDSexp.

## Methods

In twenty-four Wistar rats (365±55g), ARDSp and ARDSexp were induced by lipopolysaccharide (LPS) administered either intratracheally (200 µg) or intraperitoneally (1,000 µg), respectively. After 24h, animals were mechanically ventilated with: tidal volume (V_T_)=6ml/kg, respiratory rate (RR)=80bpm, positive end-expiratory pressure (PEEP)=0 cmH_2_O, and fraction of inspired oxygen (FiO_2_)=0.4. Baseline data were collected to evaluate if ARDSp and ARDSexp animals presented similar degree of lung damage. Rats were then randomly assigned to be mechanically ventilated with VV or CV. VV was applied on a breath-to-breath basis as sequence of randomly generated V_T_ values (n = 600; mean V_T_ = 6 ml/kg), with 30% of coefficient of variation. After randomization, all animals were ventilatedfor 1h, and lungs were removed for histology.

## Results

Variable ventilation led to decreasedrespiratory system and transpulmonary pressures in ARDSp (p< 0.05), but not in ARDSexp. Furthermore, in ARDSp, the increment of lung resistance along 1h was minimized in VV compared to CV (7% *vs*. 31%, respectively). Oxygenation increased in VV and CV regardless of ARDS etiology. Nevertheless, animals that underwent VV presented a higher percentage of increase in arterial oxygen partial pressure compared to those that underwent CV (ARDSp, 50% *vs*. 26%; ARDSexp, 100% *vs*. 53%, respectively). In ARDSp, but not in ARDSexp, there was a decrease in collapsed areas in VV compared to CV (p< 0.001).

## Conclusions

In the present model of ARDSp and ARDSexp, oxygenation improved independent of ARDS etiology, however, respiratory system and transpulmonary pressures as well as collapsed areas reduced only in ARDSp. Therefore, the morphofunctional improvement in animals ventilated with VV is dependent on ARDS etiology, and this achievement could be related to better recruitment.
